# The complete mitochondrial genome of the common pine vole *Terricola subterraneus* (Arvicolinae, Rodentia)

**DOI:** 10.1080/23802359.2019.1687026

**Published:** 2019-11-12

**Authors:** Olga V. Bondareva, Natalia I. Abramson

**Affiliations:** Zoological Institute Russian Academy of Sciences, Saint-Petersburg, Russia

**Keywords:** Mitochondrion, common pine, *Microtus subterraneus*, complete genome

## Abstract

In this paper, we report the complete mitochondrial genome of the common pine vole *Microtus (Terricola) subterraneus*, which was sequenced for the first time using Illumina next-generation sequencing (NGS) technology. The total length of the mitogenome was 16,398 bp and contained 12S, 16S rRNAs, 22 tRNAs, 13 protein-coding genes, and a 883 bp D-loop in the characteristic arrangement of subfamily Arvicolinae, Rodentia. Overall base composition of the complete mitochondrial DNA is A (33.0%), C (26.5%), G (13.4%), and T (27.0%), respectively. Phylogenetic analysis of mitochondrial genomes showed a classic taxon pattern, identified using individual phylogenetic markers.

The common pine vole *Terricola subterraneus* (Sèlys-Longchamps, 1836) is a common species of forest edge and deforested habitats, also of alpine dock forbs. Its distribution range stretches from Atlantic coast through Central to Eastern Europe (Musser and Carleton 2005), also isolated populations are reported from vicinities of Saint-Petersburg (Zagorodnyuk 1989). However, it is one of the least studied rodent species of voles within the East Europe. The specimens of *T. subterraneus* were trapped in National Natural Park ‘Homilshanski Forests’, near Gaidary village, Zmiev district, Kharkiv region, Ukraine, GPS coordinates: 49.611254, 36.323785

The tissue and DNA of the specimens were deposited in the Theriology collection of the Zoological Institute RAS, Saint Petersburg, Russia, under the number 4875. Total genomic DNA was isolated from the muscle tissue stored in 96% alcohol and paired-end sequencing (2 × 100 bp) was performed in an Illumina HiSeq sequencer in Genomics Core Facility of Skolkovo Institute of Science and Technology. Bases with a quality score lower than 20 were removed from sequence reads, and then adaptors were discarded using Trimmomatic (Bolger et al. 2014). Remaining reads were assembled using SPAdes version 3.10.1 (Bankevich et al. 2012). The assembled mitogenome sequence was annotated using the MITOS (Bernt et al. 2013), annotations were corrected manually.

The complete mitogenome of *T. subterraneus* is a closed-circular molecule of 16,398 bp in length (GenBank accession No. MN326850), and contains the typical set of 13 protein-coding genes (PCGs), 2 ribosomal RNA genes (rrnL and rrnS), 21 transfer RNA genes (tRNAs), and a putative control region. The gene order and organization of *T. subterraneus* are consistent with another rodents mitochondrial sequences. The nucleotide composition is significantly biased (A, C, G, and T was 33.0%, 26.5%, 13.4%, and 27.0%, respectively) with G + C contents of 40.0%. The GC-skew of this genome was −0.33. Nine genes (nad6 and eight tRNAs) were oriented on the reverse direction, whereas the others were transcribed on forward direction.

The *T. subterraneus* mitogenome harbors a total of 73 bp overlapping sequences in 10 regions. The longest overlap is 43 bp in length, and located between atp8 and atp6. All tRNAs have the typical cloverleaf structure, which are similar to those reported in most animal mitogenomes. The initial codons for 13 PCGs of *T. subterraneus* were the canonical putative start codons ATN (ATG for cox1, cox2, atp8, atp6, cox3, nad4l, nad4, nad5, nad6 and cob; ATT for nad3 and nad2; ATA for nad1). The typical termination codon (TAA or TAG) occurs in 11 PCGs. The remaining cox3, nad3 terminated with TTA.

Based on the full mitochondrial genome alignment, the neighbour-joining method was used to construct the phylogenetic relationship of *T. subterraneus* with other voles within subfamily with annotated mitochondrial genome. The dwarf hamster *Cricetulus griseus* Milne-Edwards, 1867 was used as an outgroup. The result showed conventional taxon pattern, identified using individual molecular markers with the position of *T. subterraneus* sister to *Microtus arvalis* sensu lato within Arvicolini tribe (Figure 1).

**Figure 1.**
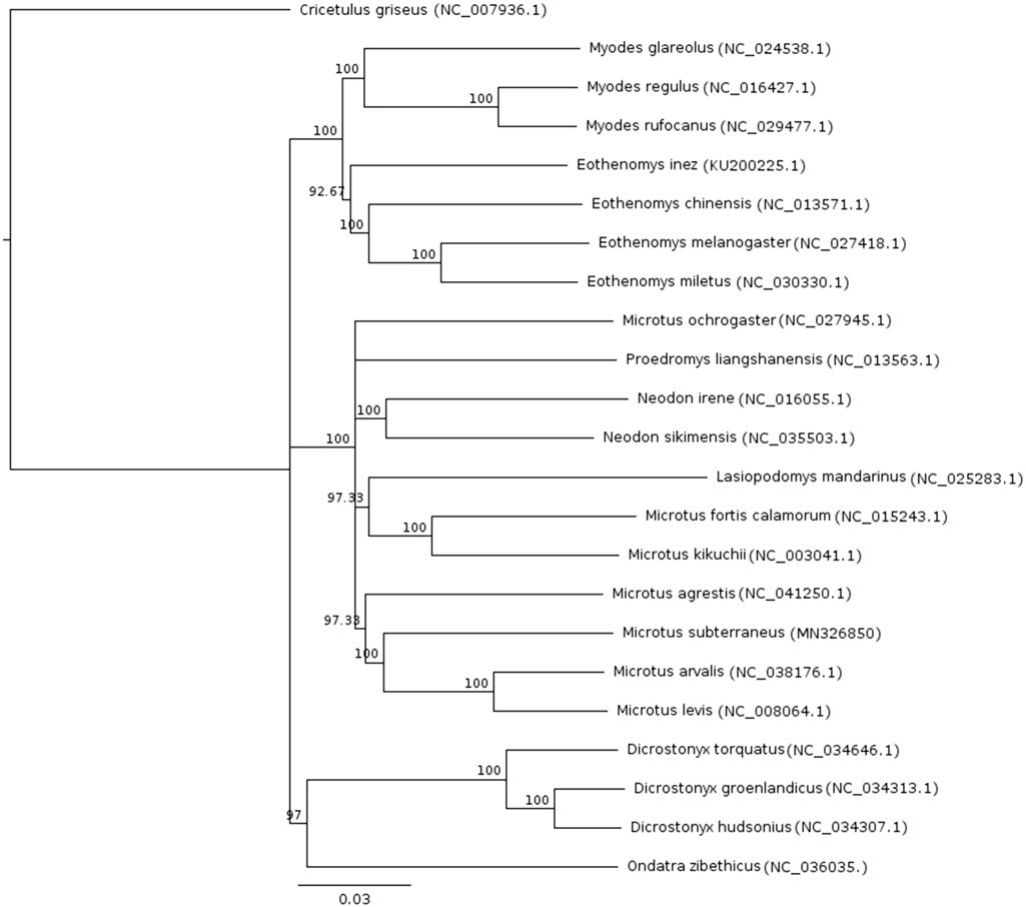
Neighbour-joining phylogenetic tree of 23 Rodent species including *Cricetulus griseus* as outgroup based on full mitochondrial alignment. Nodal numbers represent bootstrap support with 500 bootstrap replicates.
